# The architecture of the gene regulatory networks of different tissues

**DOI:** 10.1093/bioinformatics/bts387

**Published:** 2012-09-03

**Authors:** Jie Li, Xu Hua, Martin Haubrock, Jin Wang, Edgar Wingender

**Affiliations:** ^1^The State Key Laboratory of Pharmaceutical Biotechnology and Jiangsu Engineering Research Center for MicroRNA Biology and Biotechnology; ^2^Department of Bioinformatics, University Medical Center Göttingen, Goldschmidtstrasse 1, D-37077 Göttingen, Germany

## Abstract

**Summary:** The great variety of human cell types in morphology and function is due to the diverse gene expression profiles that are governed by the distinctive regulatory networks in different cell types. It is still a challenging task to explain how the regulatory networks achieve the diversity of different cell types. Here, we report on our studies of the design principles of the tissue regulatory system by constructing the regulatory networks of eight human tissues, which subsume the regulatory interactions between transcription factors (TFs), microRNAs (miRNAs) and non-TF target genes. The results show that there are in-/out-hubs of high in-/out-degrees in tissue networks. Some hubs (strong hubs) maintain the hub status in all the tissues where they are expressed, whereas others (weak hubs), in spite of their ubiquitous expression, are hubs only in some tissues. The network motifs are mostly feed-forward loops. Some of them having no miRNAs are the common motifs shared by all tissues, whereas the others containing miRNAs are the tissue-specific ones owned by one or several tissues, indicating that the transcriptional regulation is more conserved across tissues than the post-transcriptional regulation. In particular, a common bow-tie framework was found that underlies the motif instances and shows diverse patterns in different tissues. Such bow-tie framework reflects the utilization efficiency of the regulatory system as well as its high variability in different tissues, and could serve as the model to further understand the structural adaptation of the regulatory system to the specific requirements of different cell functions.

**Contact:** edgar.wingender@bioinf.med.uni-goettingen.de; jwang@nju.edu.cn

**Supplementary information:** Supplementary data are available at *Bioinformatics* online.

## 1 INTRODUCTION

There are some hundred of cell types in the human body harboring the same genome, but showing quite diverse behaviors in morphology and biological functions. This suggests that the cells of different types are developed by eliciting different gene regulatory networks, all encoded in the human genome (Ben-Tabou de-Leon and Davidson, 2007). This raises a number of questions: what is the mechanism that governs the activation of different gene regulatory networks? Are the gene regulatory networks accounting for the different types of cells designed under a same principle or architectural framework? What kinds of differences in regulatory networks contribute to the diversity of cell types? These are key questions in understanding the mechanisms underlying the specific functions of different cell types and are essential for cell regeneration that closely relates to the treatment of tissue damage and injury involved in various diseases.

The gene regulatory network of a cell is supposed to contain the comprehensive regulatory information governing the gene expression in this cell. In previous works, the gene regulatory network mainly referred to the transcriptional network which depicts the regulations at transcriptional level (Babu *et al.*, 2004; Cosentino Lagomarsino *et al.*, 2007; Yu *et al.*, 2003). Nevertheless, the microRNA (miRNA), a small RNA that negatively regulates the gene expression at post-transcriptional level by binding to the mRNA sequences of its target gene, was found at the end of the 20th century (Lee *et al.*, 1993; Wightman *et al.*, 1993). So far, >20 000 microRNAs of different species have been identified (Griffiths-Jones *et al.*, 2008), and many useful databases and effective tools that are used to predict the targets of miRNAs have been developed (Krek *et al.*, 2005; Lewis *et al.*, 2003; Sethupathy *et al.*, 2006). This gives the possibility to construct a more comprehensive map of gene regulatory networks for certain cell types by integrating transcriptional and post-transcriptional regulations of gene expression.

Nowadays, there are several studies on the gene regulatory networks covering both the transcriptional and post-transcriptional regulation of genes. For example, Pilpel and co-workers constructed a global miRNA–TF regulatory network for mammalian and studied the combinatory regulations between TFs and miRNAs according to the local and global architecture of the network (Shalgi *et al.*, 2007). Qian *et al.* investigated the biological functions of miRNAs by pursuing the miRNA motif profiles in the miRNA-TF regulatory network (Yu *et al.*, 2008). Gersten *et al.* developed an integrated strategy to construct and analyze the gene regulatory network from high-throughput sequencing data (Cheng *et al.*, 2011). However, all of these works are focused on the genome-wide level. Studies about the regulatory networks of specific tissues and their design principles are largely missing.

Here, we constructed the large-scale gene regulatory networks of eight human tissues and investigated their design principles from the perspective of three levels, i.e. the local structure of vertices (i.e. degrees), the small circuits (i.e. network motifs) and the assembly of small circuits. Our results show that the tissue-specific regulatory networks (TRNs) constructed and analyzed here contain hub nodes, the specific features of which may vary considerably among the tissues investigated. The different TRNs also vary significantly with regard to the composition of topological motifs, the instances of which are organized in a bow-tie structure. The bow-tie structures of the eight TRNs investigated classify them into symmetric, input-oriented and output-oriented networks.

## 2 METHODS

### 2.1 Network construction

We constructed the TRN for each tissue in the following two steps. First, a reference network was constructed by predicting the gene regulatory relations between TFs, miRNAs and non-TF protein genes throughout the whole human genome. Then, the TRN for a certain tissue was built by extracting the TFs, miRNAs and non-TF protein genes that are known to be expressed in the respective tissue as well as regulatory relationships between them from the reference network.

TF targets were predicted by identifying conserved potential TF-binding sites (TFBSs) within the 1-kb upstream regions of all human genes (RefSeq annotation). This was done using the position weight matrix library of the TRANSFAC database (version 2009.4) (Wingender, 2008) and the associated program Match (Kel *et al.*, 2003). The highest default thresholds (‘minFP’) were used to minimize false positives. These predicted TFBSs were filtered for those conserved across five mammalian species (human, mouse, dog, cow and opossum), based on RefSeq annotation of UCSC hg18 (http://genome.ucsc.edu/index.html), which reduced further the number of false positives. As for miRNA clusters, potential TFBSs were searched for in the 10-kb upstream to the start point of the first miRNA in the cluster. In addition, we predicted the interactions starting from miRNAs by the three tools of Targetscan (Lewis *et al.*, 2003), Pictar (Krek *et al.*, 2005) and Tarbase (Sethupathy *et al.*, 2006). The union of the results predicted by these three tools was taken to give a comprehensive regulatory network. Replacing the union of the results with the results of Tarbase, which are verified by experiments, did not affect our conclusion.

The gene expression data for TF and non-TF protein genes that we used to build the different TRNs were collected from UniGene (Sayers *et al.*, 2012) and CGAP (Lash *et al.*, 2000), whereas the miRNA expression data were obtained from a report published in 2007 (Landgraf *et al.*, 2007).

### 2.2 In-/out-hubs and specificity index

The in-/out-hubs are defined in a statistical way. We firstly generated 2000 equivalent random networks, where the number of vertices and arcs are kept but the arcs are randomly distributed among distinct vertex pairs. The in-/out-hubs are defined as those vertices whose in-/out-degrees are significantly higher (*P* < 0.01) than that of the same vertices in the random networks.

The tissue specificity of an in-/out-hub is characterized by the specificity index (SI), written as
(1)
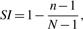

where *N* is the total number of tissues, and n is the number of tissues where the gene acts as hub. Obviously, SI varies within the range of 0 to 1. It equals to 1 when the gene is a hub in only one TRN, whereas it equals to 0 when the gene is the hub in all of the TRNs.

### 2.3 Standard deviation of hubs (*σ_RF_*)

The 2000 random cases were generated by firstly collecting all the in-/out-degree values of a certain type of genes and then randomly redistributing these degree values to the genes of the type. Each random case contained a set of eight TRNs where the in-/out-degree values of each type of genes are redistributed. The in-/out-hubs in each random case were identified (see above), and the specificity indices of the hubs are calculated in the same way as in the real case.

The standard deviation of hubs pertaining to a certain specificity index was calculated in the following way:
(2)
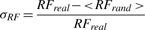

where *RF_real_* is the relative frequency of hubs in the real case, and <*RF_rand_* > is the average frequency in the random cases.

### 2.4 Motif scanning

The three-vertex motifs were computed with the tool FANMOD (Wernicke and Rasche, 2006). The equivalent random network that was used to calculate the over-represented significance of motifs was constructed by keeping the in-/out-degree of every vertex but switching the connected vertices. For every TRN, 1000 random networks were generated. Three criteria are used to identify a motif: (i) the occurrence should be >5; (ii) the *P*-value should be <0.05 and (iii) the *Z*-score value should be >2.2.

### 2.5 Statistical testing on genes in CM and TS motif instances

#### 2.5.1 Molecular component

The mean percentages of TF, miRNA and non-TF in the non-core TS/core/non-core CM gene sets were calculated by averaging the corresponding values through eight TRNs.

#### 2.5.2 Average degree

The average degree within any set of genes in motif instances is defined in the following way:
(3)
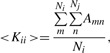

where *N_i_* is the total number of genes in set *i*, and *A_mn_* is the element of the adjacency matrix that represents the regulation from gene m to gene n in section *i*. It equals 1 when there is a regulation from gene *m* to *n*, otherwise it equals 0. The average degree from set *i* to *j* is written as
(4)
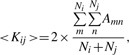


#### 2.5.3 Statistical testing

The statistical testing on the percentages of molecular components and average degrees were made by firstly generating 5000 random cases where the genes were randomly re-distributed into gene sets with the gene number in each gene set and the connections between genes being kept. The significance of the percent/average degree in the real case was evaluated by counting the probability where the real value was larger/smaller than the value in random cases.

## 3 RESULTS

### 3.1 TRN and the general property

The TRNs for the eight tissues, i.e. brain, testis, ovary, liver, spleen, heart, kidney and prostate, were constructed on the basis of a ‘reference’ network. The reference network comprises all possible regulatory interactions of TFs and miRNAs with their target genes, which may be TF, miRNA and non-TF genes. These interactions were predicted by sequence analyses throughout the human genome, in order to identify potential TFBSs and miRNA target sequences (see Section 2). During the construction of the reference network, the information whether the interacting genes are expressed in the same tissue is ignored. In contrast, the TRNs take into account the expression of genes in different tissues. They are built by extracting the TFs, miRNAs and non-TF protein-coding genes that are expressed in the tissue as well as their regulatory relationships from the reference network (see Section 2). The size of the eight TRNs varies in a broad range of <3000 to >8000 vertices (genes), and from 16 666 to 63 371 edges (interactions) (Supplementary Table 1.1)

### 3.2 Degree distribution and switches of in-/out- hubs

**Fig. 1.**
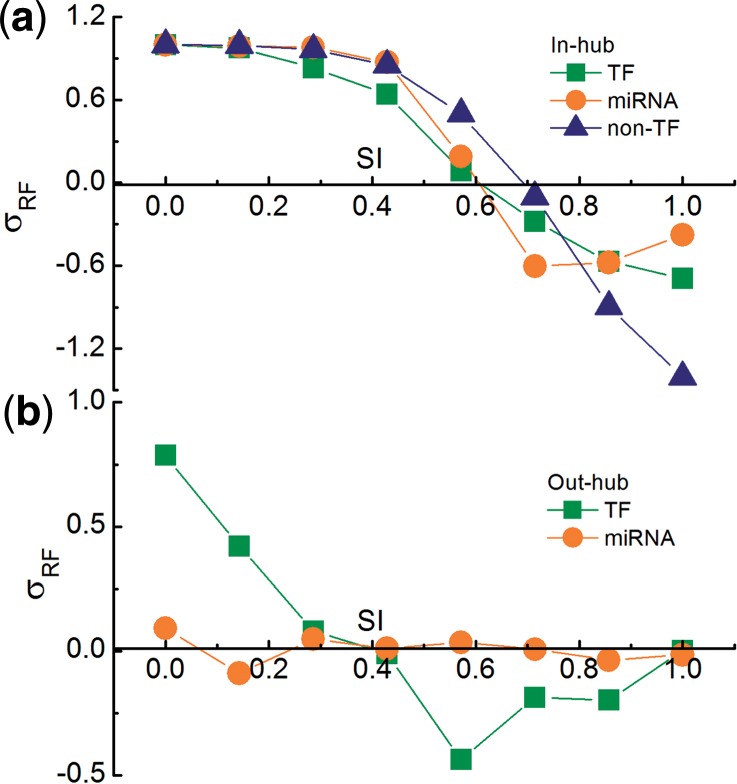
Standard deviation of relative frequency (*σ_RF_*) of hubs in real relative to random networks versus SI; (**a**) for in-hubs; (**b**) for out-hubs

For an initial characterization of the reconstructed networks, we studied the inverse cumulative in-/out-degree distributions of TFs, miRNAs and non-TFs separately in every TRN. In nearly all degree distributions examined (Supplementary Figs. 2.1–2.5), it was obvious that most of the vertices had a very low degree, whereas there were a few highly connected nodes. This suggested the presence of in-/out-hubs. They could be identified by selecting those vertices with significantly higher degree than they received in the equivalent random networks (see Section 2). There are totally 47 (or 226) miRNAs, 323 (or 180) TFs and 3631 (or 0) non-TFs that act as the in- (or out-) hubs at least in one TRN. A parameter SI (see Section 2) has been defined to scale the tissue-specificity of hubs. The closer the SI is to 1, the higher the tissue specificity of the hub is. The standard variations of the relative frequency (Swirski *et al*., 2009) of hubs (*σ_RF_*) compared to that of the random cases where the relations between the genes and their degrees are disassociated (see Section 2) are plotted against the SI in Figure 1. The *σ_RF_* for in-hubs decreases monotonically from the positive area to the negative area with increasing SI (Fig. 1a). This suggests that in-hubs tend to be shared by several tissues. TF out-hubs also have the tendency to be shared by multiple tissues because the *σ_RF_* of TF out-hubs turns from the positive to the negative as the increase of SI (Fig. 1b). However, the *σ_RF_* of miRNA out-hubs fluctuates around zero.

**Fig. 2.**
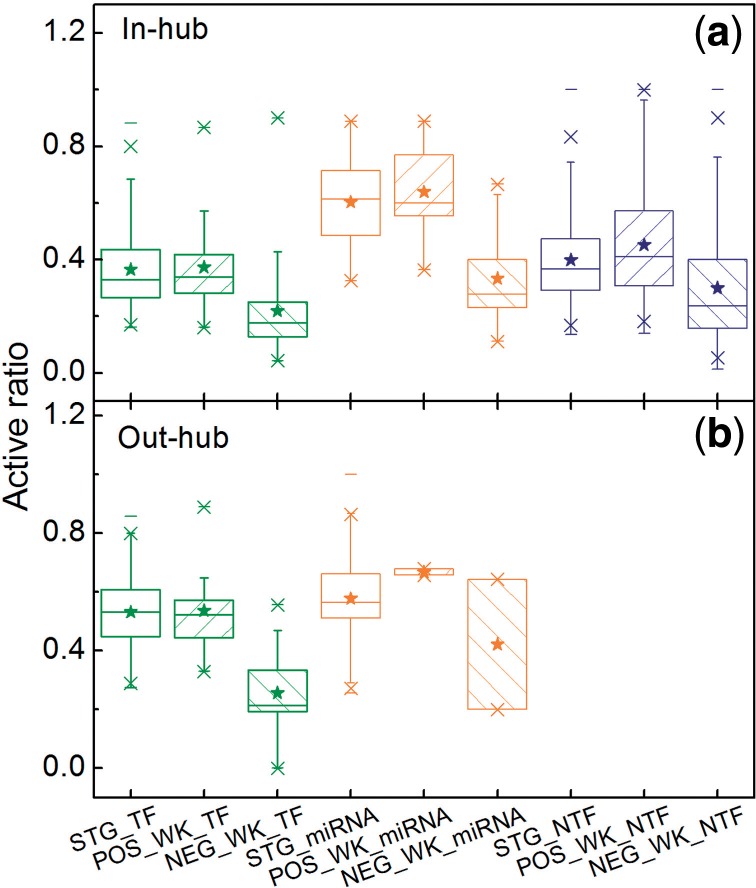
Box plots of active ratio (i.e. the active connections among all the possible connections pertaining to them in the reference network) (**a**) for in-hubs; (**b**) for out-hubs. STG_TF/STG_miRNA/STG_NTF refers to strong TF/miRNA/non-TF hubs, POS_WK_TF/POS_WK_miRNA/POS_ WK_NTF to the positive case that a weak TF/miRNA/non-TF hub acts as a hub, and NEG_WK_TF/NEG_WK_miRNA/NEG_WK_NTF the negative case that a weak TF/miRNA/non-TF hub is not a hub anymore

The majority of in-/out-hubs (90%/90% for TF in-/out-hubs, 81%/32% for miRNA in-/out-hubs, and 95% for non-TF in-hubs) are expressed in multiple TRNs. However, they are not always hubs in every tissue they are expressed in. Some hubs (strong hubs) maintain a high ratio of the active connections among all the possible connections pertaining to them in the reference network (i.e. active ratio), and therefore keep their hub status in all the tissues where they are expressed (Fig. 2a and b). The others (weak hubs) decrease their active ratio in some tissues (see the negative cases of weak hubs comparing to the positive cases in Fig. 2a and b) and switch off their hub status in some of the tissues.

### 3.3 Basic co-regulation circuits, CM and TS motifs

Network motifs are small connectivity patterns that are statistically overrepresented in a network (Shen-Orr *et al.*, 2002). We searched for three-vertex motifs in all the TRNs to study the co-regulations of and by TF and miRNA in tissues (see Table 1 and Section 2). It is seen that most of the loop motifs are feed-forward loops (Table 1). Three-vertex feedback loops, which are known to be basically absent from transcription networks (Goemann *et al.*, 2009; Shen-Orr *et al.*, 2002), were found here only as parts of complex loops which contain both the feed-forward and feedback patterns due bidirectional control (a ‘dyad’) between a miRNA and a TF vertex (‘CMPLX’ in Table 1). The participation of miRNAs additionally introduces some feedback into some motifs which helps with the fine-tuning and stability of gene regulations.

**Table 1.** Motifs in TRNs

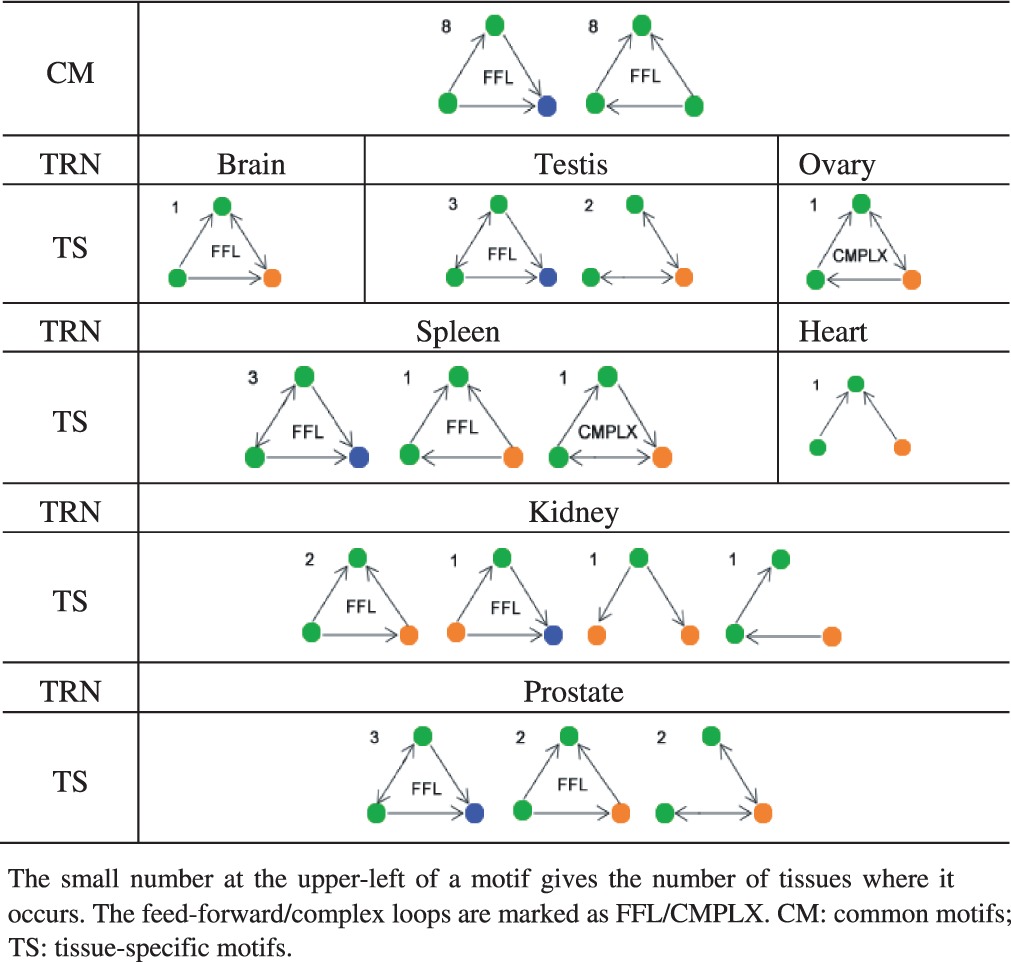

Comparing the motifs of different TRNs, we can distinguish between those that are shared by all the TRNs (common, or CM, motifs) and those that specifically occur in few tissues [tissue-specific (TS) motifs]. As shown in Table 1, there are two CM and nine TS motifs. Both CM and the majority of the TS motifs are feed-forward loops. There are no miRNAs included in the CM motifs, whereas almost all TS motifs contain at least one miRNA; the only exception is the FFL consisting of two TF and one non-TF gene, which occurs in three of the eight tissues studied (Table 1). This means that miRNAs greatly contribute to the tissue specificity of combinatory regulations, which may be related to the diversity of regulatory patterns across tissues. This is equivalent to the suggestion that the combinatory regulations at the transcriptional stage tend to be conserved across tissues, while the ones involved in the post-transcriptional stage are switching.

### 3.4 Bow-tie structure of CM–TS assembly

To study potential higher order structures of our TRNs, we collected all the instances of CM and TS motifs and traced the structural organization of the CM part (i.e. the part formed by all the instances of CM motifs) and the TS part (all the instances of TS motifs) in every TRN Since there are no TS motifs in the liver TRN, we started the study with the TRNs of the other seven tissues. In general, the CM and TS instances together form a CM-TS block in each of the seven TRNs, connected through genes that are shared by CM and TS motifs (termed as core genes). These core genes together with non-core CM genes and non-core TS genes constitute a tripartite structure of each TRN, except of the liver TRN that only contains non-core CM genes.

**Fig. 3.**
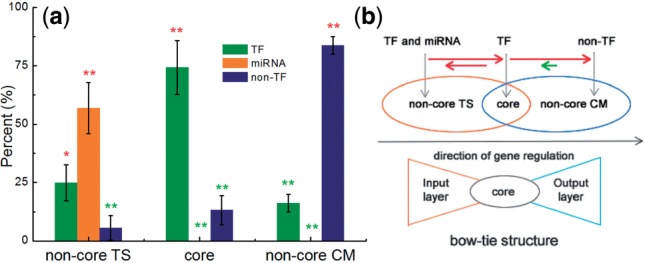
Bow-tie structure of motif instances. (**a**) Distribution of TFs, miRNAs and non-TFs; (**b**) sketch of bow-tie structure. In (a), significance (*P* < 0.05) compared with randomized networks is indicated by one or two (significant in some or all tissues) asterisks; red asterisks: over-, green asterisks under-representation. The horizontal arrow in (b) represents the degrees between three gene sets. Its length is proportional to the value of degree. The red/green coloring indicates that the degree is significantly higher/lower than in the random cases

We found that >75% of core genes and 80% of non-core CM genes, respectively, are TFs and non-TFs (Fig. 3a). Such high population of TFs/non-TFs is statistically significant when compared to the random case where the genes were randomly picked as core, non-core CM and non-core TS genes (*P* < 0.05, see Section 2), suggesting TF and non-TF, respectively, are the dominating genes of core and non-core CM gene set. No miRNAs are found in the core and non-core CM gene sets. The miRNAs are significantly enriched in the non-core TS set (*P* < 0.05). In some TRNs (i.e. heart and prostate), TFs are also enriched in the non-core TS gene set. The sum of TF and miRNA percent is >80% on average (Fig. 3a). This indicates that TF and miRNA are the major components of the non-core TS gene set.

The average degrees within and between the three gene sets were also calculated and compared to the random cases (Fig. 3b and see Section 2). We found that the average degree from non-core TS section to core section and that from core section to non-core CM section are both significantly elevated. Of the ‘backwards’ connections, those from non-core CM to core are significantly decreased, while those from core to non-core TF are also enhanced, to but a lesser extent (about half) than the opposite direction. These results suggest a regulation flow from non-core TS section to non-core CM section through the core section and that the non-core CM section is the terminal part of the regulation flow. The CM-TS block in TRNs is thus organized as a bow-tie structure (Fig. 3b), which is composed of an input (non-core TS), core and output (non-core CM) layer (Csete and Doyle, 2004).

### 3.5 Patterns of bow-tie structure

**Fig. 4.**
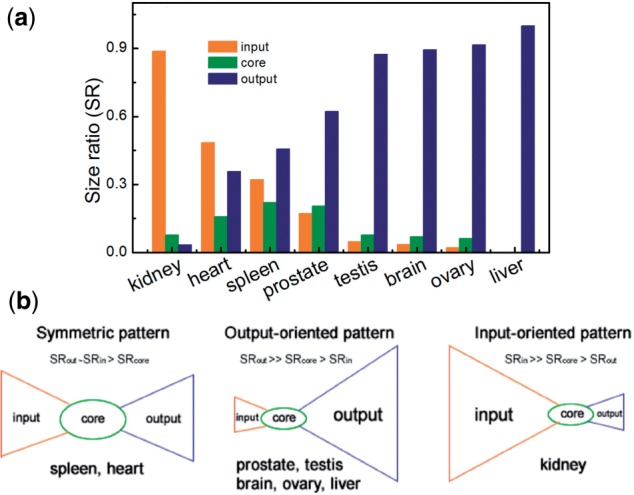
Three patterns of bow-tie structure. (**a**) Size ratio of input, core and output layer in eight tissues; (**b**) illustration of patterns

Although the bow-tie architecture of the CM-TS block exists throughout the TRNs for various tissues, distinctive patterns are found as considering the size ratio (SR) of the input, core and output layer (Fig. 4a). In the order kidney–heart–spleen–prostate–testis–brain–ovary–liver, the proportion of the miRNA-dominated input layer decreases from 0.88 down to 0, whereas that of the output layer where the non-TF genes are predominant increases from 0.03 to 1.0. In this order, the core component with tions may also found a grouping of the studied tissues into those (i) with output-dominated TRNs (prostate, testis, brain, ovary and liver), where SRinput < SRcore << SRoutput, (ii) with a symmetric TRN (heart, spleen), where SRinput ≅ SRoutput > SRcore and (iii) with an input-dominated TRN (kidney), where SRinput >> SRcore > SRoutput (Fig. 4b).

## 4 DISCUSSION

The networks constructed here comprise edges that, to the best of our knowledge, are predicted by the most reliable approaches available. As for the miRNA–target relations, we took the union of the results of a number of predictors (see Section 2). Concerning the TF-target connections, we are confident that we successfully minimized the false-positive rate, since a high percentage of the predicted edges could be recovered from available high-throughput datasets as well (Haubrock *et al.*, in preparation). The trade-off of this attempt, however, certainly was a considerable false-negative rate. It should thus be noted that the networks presented here are only based on the subset of TFBS with highest affinity and conservativity, to which we attribute the property of being ‘seed’ sites (in preparation). These seed sites are assumed to play a particular role in flagging promoter regions, and thus would nicely coincide with the recently published concept of ‘pioneer factors’ (Zaret and Carroll, 2011).

As a first attempt to globally characterize a network, the degree distribution is usually analyzed. The TRNs constructed here show a kind of mixed power-law/exponential (or ‘broad-scale’ distribution, or ‘truncated scale-free’, with their power-law exponents in the range of 0.8–2.4) degree distribution, which may be more on the power-law side in case of all in-degree distributions, or more on the exponential side for the miRNA out-degree distribution. In any case, however, we observed that most nodes of our TRNs exhibit a low connectivity, while very few nodes show a very high degree. We therefore felt that the concept of hub nodes is applicable to the networks constructed and studied here.

Different functional types of hubs can be distinguished in the TRNs, depending on the nature of the genes and whether they are in- or out-hubs. The TF/miRNA in-hubs (e.g. E2F3 and SOX2 in brain, testis and prostate, RUNX2 in heart and liver and hsa-miR-124 in brain and prostate) act as the central processors, receiving and integrating a great many of regulatory input from upstream regulators and re-distribute it to the downstream genes, whereas the TF/miRNA out-hubs (e.g. ATF6, MYC, hsa-miR-29A and hsa-let-7B in all the eight tissues) act as central coordinators because they regulate large numbers of targets. Interestingly, some TFs and miRNAs are both in- and out-hubs (e.g. RUNX3 in testis, ovary, spleen and prostate, BCL6 and hsa-miR-22 in all the eight tissues), acting as the central processors as well as central coordinators. In contrast, the non-TF in-hubs (e.g. MAPK4 in brain, prostate and liver, AK2 in all of the eight tissues) may be central executors in the downstream biological processes since they are the terminal genes in regulation whose expression level is finely controlled by complex regulation. In contrast to the majority of genes in the TRNs (Supplementary Fig. 3.1), the in-/out-hubs show a clear tendency to be shared by multiple tissues. This may relate to an efficient utilization of the gene regulatory system.

There shows two kinds of hubs according to hub behaviors in different tissues. One type preserves its hub status in all the tissues they are expressed in (strong hubs), whereas hubs of the other type switch into a non-hub in some of the tissues where they are expressed (weak hubs). More miRNA than TF hubs, in particular almost all miRNA out-hubs, are strong hubs (Supplementary Fig. 3.2). Thus, the local structures around miRNA hubs are more conserved across tissues than those of TF hubs. Since out-hubs are more frequently strong hubs than in-hubs, central coordinators are more preserved through different tissues than central processors, which account for the integration of largely TS regulations. In contrast to non-TF strong hubs, TF and miRNA strong hubs more often exhibit different neighborhoods in the different tissues, underpinning again the flexible re-usability of the regulatory machinery (Supplementary Figs 3.3 and 3.4). Nevertheless, no distinctive difference in this respect between TF and miRNA strong hubs is found.

The network motifs give the basic co-regulation circuits of TFs and miRNAs (Shalgi *et al.*, 2007; Yu *et al.*, 2008). Our study focuses on three-vertex motifs since they have the minimal size to provide an integrative regulation. The FFL, which is the dominating motif in the transcriptional networks (Goemann *et al.*, 2009; Shen-Orr *et al.*, 2002), is also the main type of combinatory regulations in our regulatory networks covering the regulations of TFs and miRNAs. This indicates the main task of the regulatory system is information-processing performed by FFLs (Shen-Orr *et al.*, 2002), and the miRNAs mainly account for the fine-tuning and stability of gene regulations.

The two common motifs shared by all tissues contain only TFs and non-TFs. They are also the fundamental motifs which are widely distributed in the transcriptional networks of various species (Milo *et al.*, 2004). The transcriptional regulation may be the more stable part in the gene regulatory system, which is more conserved during the development of tissues as well as the evolution of species. The TS motifs are special for one or several tissues and almost all of them contain a miRNA component, indicating that the post-transcriptional regulations are mainly responsible for the diversity of tissue specificities. Such diversity in the post-transcriptional stage is not simply related to the high tissue specificity of miRNAs in expression. It also relates to the connecting patterns that the co-regulation of miRNA and TF shows in different tissues. This is because motif is a structural concept which does not consider the identities of specific genes that are involved in. There is the case that a motif is conserved across tissues, while the genes that constitute the motif are quite different in different tissues.

From the specific interactions between common (Krek *et al*., 2005) and TS motif instances, we concluded that the motif instances are organized in a bow-tie structure with specific enrichment of miRNA, TF and non-TF genes in the input, core and output layer (Supplementary Fig. 5.1). The bow-tie structure is an architecture that is capable of adapting the highly fluctuating environment of biological system and mediates trade-offs among efficiency, robustness and evolvability (Csete and Doyle, 2004). Its three-layer structure allows the fanning-in and fanning-out of large-scale regulatory flow in a highly efficient and robust way. The variability of input and output layers also provides high evolvability to satisfy diverse functional requirements in different tissues. For example, the bow-tie structures of spleen and heart represent a symmetric pattern where the sizes of input and output layers are comparable, whereas the structures of kidney (or prostate, testis, brain, ovary and liver) behave in an input-oriented (or output-oriented) pattern where the size of the input (or output) layer is distinctively larger than the other two layers. The input layer of the liver TRN is not recognizable. The bow-tie pattern of each tissue is generally related to its physiological function, for instance, all of the three reproductive tissues (i.e. prostate, testis and ovary) show the same pattern. This may be due to the fact that the tissues performing relevant physiological functions are usually similar in the expression profile of mRNA and miRNA (Liang *et al.*, 2007; Shyamsundar *et al.*, 2005), which consequently results in the high similarity of their TRNs, and thus the similarity of the bow-tie patterns. In particular, it is interesting that brain is also clustered together with testis. This is probably related to the finding that these two tissues are of similar mRNA and miRNA expression profiles, although the reason for this similarity is still unclear (Liang *et al.*, 2007; Shyamsundar *et al.*, 2005). Both tissues have also been shown in rat to express the highest numbers of TS miRNAs (Linsen *et al.*, 2010). The mRNA and miRNA expression profiles of spleen and heart are distinct. However, it is known that the spleen in mouse serves as a reservoir for immune cells (monocytes) that function in repairing the heart after myocardial infarction (Swirski *et al.*, 2009). Such connection implies that there could be some essential regulators or genes shared by spleen and heart which contribute to the similarity of their bow-tie pattern. Besides, the kidney TRN is the only one that exhibits an input-oriented pattern, in which the input layer enriching the combinatory regulations between TFs and miRNAs is of a particularly large size. This may be related to the fact that kidney is an organ that plays essential regulatory roles in the urinary system, as well as in the homeostasis of the internal environment (via adjusting the balance of the electrolytes and acid–base etc.) and regulating blood pressure (via maintaining salt and water balance).

*Funding*: The project was partially funded by the European Union FP7 (LipidomicNet, agreement no. 202272). This work was also supported by grants from the National Natural Science Foundation of China (30890044) and the International Bureau of the Federal Ministry of Education and Research (CHN08/031).

*Conflict of Interest*: none declared.
